# An Internet-Based Self-Help Intervention for Skin Picking (SaveMySkin): Pilot Randomized Controlled Trial

**DOI:** 10.2196/15011

**Published:** 2019-09-20

**Authors:** Christina Gallinat, Markus Moessner, Holger A Haenssle, Julia K Winkler, Matthias Backenstrass, Stephanie Bauer

**Affiliations:** 1 Center for Psychotherapy Research University Hospital Heidelberg Heidelberg Germany; 2 Department of Dermatology University Hospital Heidelberg Heidelberg Germany; 3 Institute of Clinical Psychology Hospital Stuttgart Stuttgart Germany; 4 Institute of Psychology Department of Clinical Psychology and Psychotherapy Heidelberg University Heidelberg Germany

**Keywords:** skin picking, excoriation disorder, dermatillomania, internet-based, self-help, cognitive-behavioral therapy

## Abstract

**Background:**

In spite of the psychosocial burden and medical risks associated with skin picking disorder, the health care system does not provide sufficient treatment for affected individuals to date. Therefore, an internet-based self-help program for skin picking was developed to offer easily accessible support for this population.

**Objective:**

This pilot study evaluated the internet-based self-help program SaveMySkin. The 12-week program is based on cognitive-behavioral therapy and contains comprehensive information and exercises, a daily supportive monitoring system, and dermatological and psychological counseling via internet chat. Primary objectives were the investigation of attitudes and expectations toward the program, intervention effects on skin picking severity, user satisfaction, adherence, and willingness to participate. Secondary outcomes included the feasibility of study procedures, adequacy of assessment instruments, effects on skin picking–related impairment, dimensions of skin picking, and general psychological impairment.

**Methods:**

A two-arm randomized controlled trial was conducted in a sample of 133 participants (female: 124/133, 93.2%; mean age 26.67 [SD 6.42]) recruited via the internet. Inclusion required a minimum age of 17 years and at least mild skin picking severity. Participants were randomly allocated to the intervention (64/133, 48.1%) or waitlist control group (69/133, 51.9%). All assessments were conducted online and based on self-report.

**Results:**

The willingness to participate was very high in the study, so the initially planned sample size of 100 was exceeded after only 18 days. Participant expectations indicate that they believed the program to be beneficial for them (131/133, 98.5%) and provide a feeling of support (119/133, 89.5%). Reasons for study participation were insufficient outpatient health care (83/133, 62.4%) and flexibility regarding time (106/133, 79.7%) and location (109/133, 82.0%). The post-assessment was completed by 65.4% (87/133) of the sample. The majority of the intervention group who completed the entire post-assessment were satisfied with SaveMySkin (28/38, 74%) and agreed that the program is an appropriate support service (35/38, 92%). On average, participants viewed 29.31 (SD 42.02) pages in the program, and 47% (30/64) of the intervention group used the monitoring at least once a week. In comparison with the control group, the intervention group displayed substantial improvements in the skin picking severity total score (Cohen *d*=0.67) and especially on the subscale Symptom Severity (Cohen *d*=0.79). No effects on secondary outcomes were found.

**Conclusions:**

This study confirms the need for easily accessible interventions for skin picking disorder and the high interest in internet-based self-help within the target population. It provides important insights into the attitudes toward online support and actual user experiences. Participant feedback will be used to further enhance the intervention. Our results point to the preliminary efficacy of SaveMySkin and may lay the foundation for future research into the efficacy and cost-effectiveness of the program in a multicenter clinical trial.

**Trial Registration:**

German Clinical Trial Register DRKS00015236; https://www.drks.de/drks_web/navigate.do? navigationId=trial.HTML&TRIAL_ID=DRKS00015236

**International Registered Report Identifier (IRRID):**

RR2-10.1016/j.conctc.2018.100315

## Introduction

### Background

Skin picking is a body-focused repetitive behavior receiving increased attention since “excoriation (skin picking) disorder” was recognized as a distinct category within the Obsessive-Compulsive and Related Disorders of the *Diagnostic and Statistical Manual of Mental Disorders, 5th Edition* (DSM-5). The core symptom of the disorder is a recurrent behavioral pattern of manipulating one's own skin (eg, scratching, squeezing, excoriating, picking), which causes skin damage including wounds, erosive skin lesions, and scars in the long term. Individuals with skin picking are not able to resist the urge or stop the behavior [1]. Skin picking disorder predominantly affects females [2-4], and its lifetime prevalence is estimated at 1.2% to 1.4% [5,6].

Skin picking symptomatology is associated with impairment on psychological, social, and physical levels: affected individuals suffer not only from wounds, infections, and scarring [7,8] but also from embarrassment, guilt, and symptoms of depression and anxiety [2,3,9,10]. Occupational interferences, academic impairment, and a financial burden due to skin picking–related costs (eg, concealing cosmetics and clothing, fees for treatment) have also been reported [9,10]. In light of the substantial psychosocial impairment and risk for chronicity [2,11], the need for affected individuals to receive timely professional treatment is evident.

However, research on interventions for skin picking disorder has been very scarce until now. A limited number of studies have investigated pharmacological and behavioral interventions [12], including habit reversal training [13,14], acceptance and commitment therapy [15], cognitive-behavior therapy (CBT) [16,17], and combined approaches (eg, acceptance-enhanced CBT) [18,19]. Noteworthy, most of these previous studies showed severe methodological shortcomings (eg, small sample sizes, lack of control conditions), and most were conducted before the official DSM-5 criteria for skin picking disorder became available. Thus, the current evidence base for treatment of skin picking disorder is rather weak. Only one study investigated an internet- and CBT-based self-help intervention for skin picking; the study reported substantial improvements in symptom severity for 63% of the sample [17]. However, the study was uncontrolled, and only 4% of the initial sample (15/372) completed the entire intervention, so the results should be interpreted with caution. So far, two meta-analyses suggest an overall beneficial effect of behavioral treatments on skin picking severity, but these studies must also be seen as preliminary due to the small number of included original studies and their limited validity [20,21]. Overall, CBT seems to be the most promising approach for the treatment of skin picking disorder so far. This is also plausible in light of the fact that behavioral interventions have demonstrated efficacy and are currently considered as the method of choice in the treatment of trichotillomania [22-24], which shows substantial overlap in clinical characteristics and a high co-occurrence with skin picking disorder [25].

The scarcity of research on skin picking disorder reflects an overall lack of awareness and knowledge of this disorder. Affected individuals barely receive adequate treatment and face manifold difficulties in finding appropriate help [9]. Internet-based interventions have the potential to improve the health care situation for skin picking disorder due to their reach, accessibility, and availability. The efficacy and cost effectiveness of internet-based treatment approaches have already been proven for other psychiatric conditions including depression, anxiety, and eating disorders [26-29]. Therefore, we considered an internet-based self-help program a promising opportunity to provide support to individuals affected by skin picking. The program is conceptualized as a stand-alone intervention in order to complement conventional health care for skin picking disorder.

### Objectives

We developed an internet-based self-help program for skin picking, and conducted a pilot randomized controlled trial (RCT) to investigate the adequacy of the intervention SaveMySkin and the feasibility of the study procedures. Primary objectives of our study were the investigation of attitudes and expectations toward SaveMySkin before randomization, intervention effects on skin picking severity, and user satisfaction. Further outcomes were program adherence (intervention use) and willingness to participate.

The feasibility of study procedures (eg, recruitment, randomization), appropriateness of applied questionnaires, effects on skin picking–related impairment, dimensions of skin picking (focused vs automatic skin picking), and general psychological impairment were investigated as secondary outcomes.

## Methods

### Study Design

This pilot study followed a two-arm randomized controlled design with a 1:1 allocation to either intervention or waitlist control group. The design is fully described in the study protocol [30]. No essential changes have been made to the study protocol after study commencement. The actual sample size exceeded the initial aim due to a very fast recruitment via internet, so the original plan to expand recruitment to dermatological clinics was not pursued.

### Participant Selection

Inclusion required at least mild self-reported skin picking severity (Skin Picking Scale–Revised [SPS-R] score ≥7 [31,32]) and a minimum age of 17 years. Sufficient German language skills, home access to the internet, a smartphone, and literacy on internet and computer use were applied as implicit eligibility criteria. Potential participants were recruited via online advertisement (eg, specific forums, support groups, university mailing lists) and at a conference for skin picking and trichotillomania. In case of interest, individuals could directly access an openly available online screening questionnaire checking for eligibility. Eligible individuals were invited to register for the study and give the required informed consent for participation. Study participation did not include any restrictions concerning additional treatment use. The use of conventional treatment was assessed as part of the final questionnaire after 12 weeks.

### Study Arms

#### Intervention

Participants randomized to the intervention group received immediate access to the internet-based intervention, SaveMySkin, for 12 weeks. The program is based on CBT techniques and consists of several modules:

- Psychoeducation: information about skin picking disorder, treatment options, and dermatological topics
- Self-management: a module with three submodules (Skills: information materials and online exercises; Tools: downloadable offline trainings; and Emotions: online exercises on emotion regulation) aiming at the reduction of skin picking behavior and the enhancement of self-management skills based on classic CBT methods like self-observation, cognitive restructuring, and behavioral strategies
- Supportive monitoring: daily support via email including a motivational message in the morning and a short monitoring questionnaire in the evening, combined with an automatically generated, tailored feedback message
- Counseling via internet chat: optional personal support in individual chat sessions with psychologists or psychological and dermatological group chats

Overall, the intervention follows a flexible and demand-oriented design. Participants were therefore expected to use the program depending on their individual needs. Recommendations on the use of certain program modules or exercises were given within chat sessions or in the monitoring feedback. Additional information on all modules of SaveMySkin is provided in Gallinat et al [30].

#### Control Condition

Participants in the control group did not have access to the intervention until the final assessment after 12 weeks. In the final questionnaire, participants in the control group were asked if they would still like to use the intervention. If this was the case, intervention access was activated, and participants could use the program for 12 weeks.

### Primary Outcomes

All assessments—scheduled at t0 (screening), t1 (baseline), t2 (after 6 weeks), and t3 (after 12 weeks)—were performed as self-report online questionnaires. Screening data (t0) and data derived from the t1 assessment (right after screening and registration) are both referred to as baseline data. A detailed plan listing all assessments and instruments can be found in Gallinat et al [30].

#### Attitudes and Expectations

Attitudes and expectations toward SaveMySkin were investigated with 10 statements rated on a 4-point Likert scale from “does not apply” to “totally applies.” In addition, participants could report further reasons for participation.

#### Skin Picking Severity

Skin picking severity was assessed with the German version of the SPS-R [31,32]. The first 4 items of the scale refer to the subscale Symptom Severity and assess the frequency and intensity of the urge to pick the skin, time spent on skin picking, and control over skin picking behavior. The other 4 items form the subscale Impairment and assess impairing consequences caused by skin picking (avoidance, interference in social and occupational life, emotional distress, skin damage). All items are rated on a 5-point Likert scale from 0 to 4 in reference to the last 7 days. In our study, a very good internal consistency with a coefficient of α=.81 was observed for the total scale (Symptom Severity: α=.72; Impairment: α=.83).

#### User Satisfaction

User satisfaction was measured with self-designed items assessing overall satisfaction criteria (eg, recommendation to others, fulfillment of expectations). Satisfaction with single modules was assessed with statements rated on a 4-point Likert scale from “does not apply” to “totally applies” (eg, “I like the idea that individual chat sessions with psychologists are offered”). Participants could also indicate “not able to evaluate.”

#### Adherence and Use

Adherence and program use were automatically documented within the program. Monitoring compliance was assessed by the number of completed monitoring questionnaires. Chat use was evaluated by the number of booked individual chat appointments and log-ins into group chats. The use of other modules and of the overall program was investigated by the number of page views per module and user as well as log-ins per user.

### Secondary Outcomes

#### Skin Picking–Related Impairment

Skin picking–related impairment was assessed with a German translation of the Skin Picking Impact Scale (SPIS) [33,34]. The 10 items are rated on a 5-point Likert scale from “not at all” (0) to “severe” (4) and refer to the past 7 days. The SPIS demonstrated an excellent internal consistency in our study (α=.94).

#### Focused Versus Automatic Skin Picking

Modes of skin picking relating to the awareness of performing the behavior were assessed with a German translation of the Milwaukee Inventory for the Dimensions of Adult Skin Picking (MIDAS) [35]. The 12 items are rated on a 5-point Likert scale from “not true for any of my skin picking” (1) to “true for all of my skin picking” (5) and form the two subscales Focused Skin Picking and Automatic Skin Picking with 6 items each. Our study revealed acceptable internal consistencies for both subscales (Focused: α=.73; Automatic: α=.69).

#### General Psychological Impairment

General psychological impairment was assessed with the Clinical Psychological Diagnosis System 38 (KPD-38) [36,37]. The scale consists of 38 items assessing psychological impairment, social problems, general physical condition, general life satisfaction, competence skills, and social support. The items are rated on a 4-point Likert scale from “does not apply” (1) to “totally applies” (4). In our sample, internal consistency of the KPD-38 total score was excellent, with a Cronbach alpha coefficient of α=.94.

### Sample Size

The sample size in this pilot trial was determined on the basis of practical considerations and questions of feasibility, as Leon et al [38] recommend for pilot studies. These considerations led to a targeted sample size of 100 participants, which was assumed to be sufficient for the investigation of the study objectives.

### Randomization

Participants were equally (1:1) randomized to one of the study groups by the software based on an a priori defined list (intervention vs waitlist control) after they completed the registration and baseline questionnaire. Randomization was stratified by gender and followed a permuted block design. The allocation sequence was produced with a computerized random number generator.

### Statistical Analysis

Descriptive statistics were used to analyze data on attitudes, expectations, user satisfaction, and program use. Continuous variables were dichotomized by splitting the Likert scale (eg, “agree” contains “applies mostly” and “totally applies”; “helpful” contains “a little helpful” and “very helpful”) to analyze data with frequencies. Efficacy was tested by mixed models. Intervention effects were analyzed as cross-level interactions (group × time). The control group was coded 0, and the intervention group was coded 1. Assessment points were coded as follows: baseline (t0, t1)=0; t2=1; t3=2. In accordance with the recommendations of Lorah [39], another run of mixed-models analyses was conducted with standardized outcome variables to calculate the Cohen *d* based on the estimated coefficient per time span (Cohen *d* = standardized coefficients of the time × group interaction * max[time]). It should be noted that one participant in each study group did not complete the entire post-assessment. The analyses regarding user satisfaction and help-seeking at t3 therefore refer to n=38 (intervention group) and n=47 (control group). Statistical analyzes were conducted with SPSS Statistics version 25.0 (IBM Corp).

### Ethical Considerations

This trial was approved by the ethics committee of the Medical Faculty of Heidelberg University and registered at the German Clinical Trials Register [DRKS00015236]. The study protocol was published before recruitment was completed [30].

## Results

### Recruitment and Participant Flow

Participant enrollment started in October 2018. The planned sample size of 100 participants was achieved after only 18 days of recruitment. Advertisement was stopped then, but due to ethical considerations, screening and registration were not closed until December 2018.

**Figure 1:**
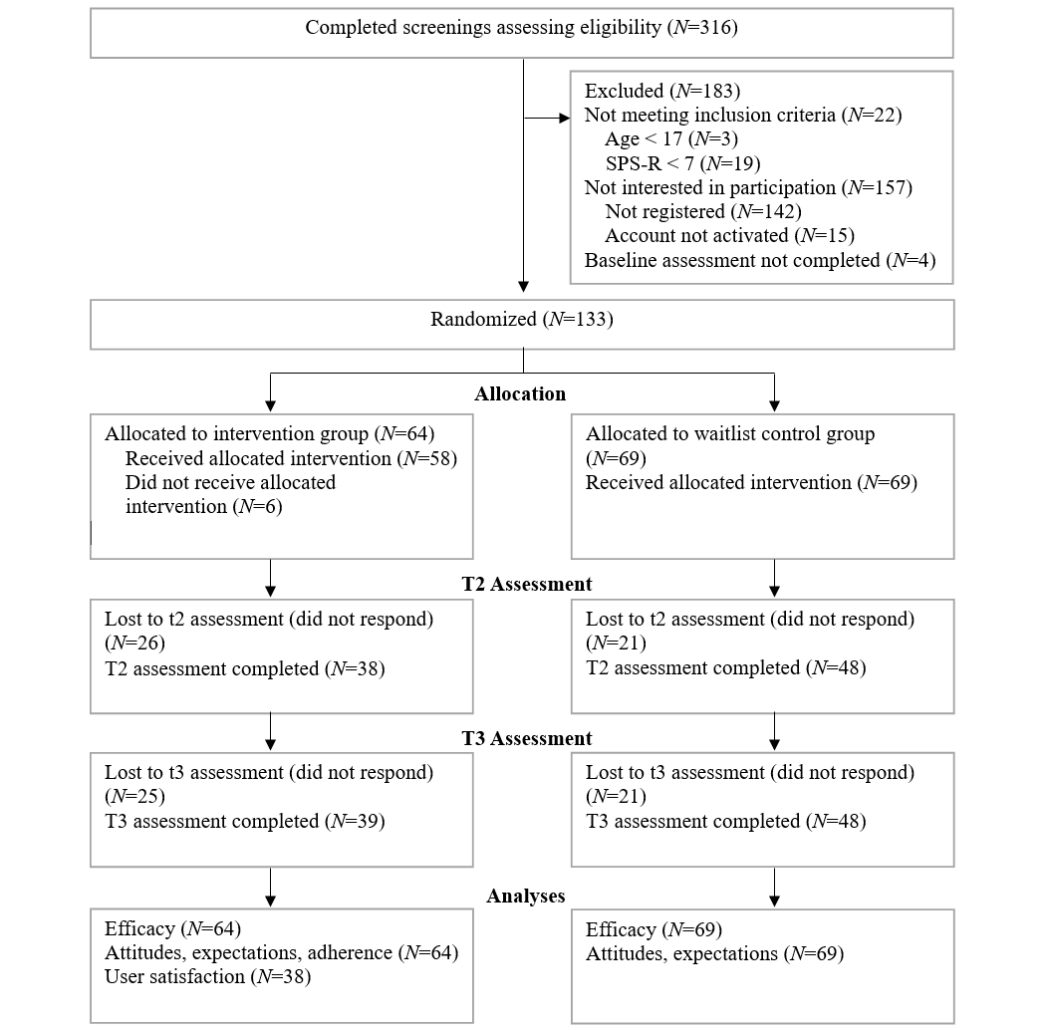
Participant flow diagram. SPS-R: Skin Picking Scale–Revised.

Out of 316 individuals who completed the screening questionnaire, 294 were eligible for study participation. More than half of this subsample registered for study participation (152/294, 51.7%), but 15 individuals did not activate their account and 4 did not complete the baseline assessment. In the end, 42.1% (133/316) of the individuals screened for eligibility completed the entire inclusion process and were therefore randomized to one of the study groups; 64 participants were allocated to the intervention group and 69 participants to the waitlist control group. Six participants in the intervention group neither logged into the program after the initial registration process nor completed any of the daily monitoring assessments, so they did not receive the allocated intervention. The response rate for the assessment at t2 (6 weeks after randomization) was 59% in the intervention group (38/64) and 70% in the control group (48/69). The final assessment (t3: 12 weeks after randomization) was completed by 61% of the intervention group (39/64) and 70% of the control group (48/69). A detailed overview of the participant flow including the number of analyzed cases for each objective is provided in Figure 1.

### Baseline Characteristics

Tables 1 and 2 show the demographic and clinical characteristics for each study group. The majority of the sample was female (124/133, 93.2%) with a mean age of 26.67 (SD 6.42) years (range 17-56).

The total sample displays high levels of skin picking severity (SPS-R scores) and skin picking–related impairment (SPIS scores). Furthermore, the participants show significant psychological impairment with regard to the KPD-38 total score (mean 2.44 [SD 0.55]), which corresponds to the 87th percentile of the norm data for women in the general population (age category: 14-34 years) [36].

Nearly one-quarter of the sample (32/133, 24.1%) was currently in psychotherapeutic or psychiatric treatment at the time of baseline assessment. Except for the SPS-R subscale Symptom Severity, the study groups did not differ in the assessed baseline characteristics.

**Table 1:** Demographic characteristics (N=133).

Characteristic		Intervention group (n=64)	Control group (n=69)	Test statistic	*P* value
Female, n (%)		61 (95)	63 (91)	0.85^a^	.36
Age (years), mean (SD)		26.19 (6.52)	27.12 (6.34)	0.83^b^	.41
**Education, n (%)**				3.07^c^	.55
	Still in school	1 (2)	1 (1)		
	Middle secondary	7 (11)	7 (10)		
	Highest secondary	29 (45)	23 (33)		
	University	27 (42)	37 (54)		
	Other	—^d^	1 (1)		
**Occupational status, n (%)**				4.54^e^	.60
	Employed	15 (23)	27 (39)		
	Trainee	5 (8)	3 (4)		
	School student	1 (2)	1 (1)		
	University student	38 (59)	35 (51)		
	Housewife/househusband	2 (3)	1 (1)		
	Retired	1 (2)	1 (1)		
	Unemployed	2 (3)	1 (1)		
**Family status, n (%)**				3.07^f^	.38
	Single	30 (47)	26 (38)		
	In a relationship	26 (41)	29 (42)		
	Married	8 (13)	12 (17)		
	Other	—	2 (3)		
Current psychotherapeutic or psychiatric treatment at baseline, n (%)		15 (23)	17 (25)	0.03^a^	.87

^a^*χ*^2^_1_.

^b^*t*_131_.

^c^*χ*^2^_4_.

^d^Not applicable.

^e^*χ*^2^_6_.

^f^*χ*^2^_3_.

**Table 2:** Clinical variables at baseline (N=133).

Characteristic		Intervention group (n=64), mean (SD)	Control group (n=69), mean (SD)	Test statistic	*P* value
**SPS-R** ^a^ **total score**		16.62 (4.33)	15.68 (4.04)	–1.30^b^	.20
	SPS-R Symptom Severity	9.94 (1.97)	9.20 (2.26)	–1.99^b^	.048
	SPS-R Impairment	6.69 (3.08)	6.48 (2.58)	–0.43^b^	.67
KPD-38^c^ total score		2.47 (0.52)	2.42 (0.57)	–0.43^b^	.67
SPIS^d^		17.73 (10.59)	18.30 (10.00)	0.32^b^	.75
MIDAS^e^ focused skin picking		18.92 (5.15)	18.48 (4.54)	–0.53^b^	.60
MIDAS automatic skin picking		17.91 (4.94)	17.99 (3.88)	0.10^f^	.92

^a^SPS-R: Skin Picking Scale–Revised.

^b^*t*_131_.

^c^KPD-38: Clinical Psychological Diagnosis System–38.

^d^SPIS: Skin Picking Impact Scale.

^e^MIDAS: Milwaukee Inventory for the Dimensions of Adult Skin Picking.

^f^*t*_119.42_.

### Attitudes and Expectations

Prior to randomization, almost all participants expected the program to be generally helpful and beneficial for them. They expected to feel supported and gain a positive effect on their well-being. More than two-thirds (83/133, 62.4%) indicated that they would like to participate due to insufficient support options within the regular health care system (Table 3).

Common reasons for participation were the flexibility of the internet-based setting regarding time (106/133, 79.7%) and location (109/133, 82.0%), expertise for skin picking (98/133, 73.7%), free counseling (97/133, 72.9%), anonymity (83/133, 62.4%), the option to contact somebody in a quick and easy way (75/133, 56.4%), and the possibility to ask questions in written form (49/133, 36.8%).

### Efficacy

In comparison to the control group, the intervention group yielded considerably higher reductions in skin picking severity and symptom severity resulting in moderate effect sizes of *d*=0.67 and *d*=0.79 (Cohen *d*, Table 4). The intervention and control group both showed improved scores on the impairment scale, but the analyses did not reveal a significant difference between the groups.

**Table 3:** Attitudes and expectations toward SaveMySkin (N=133). Answers on the 4-point Likert scale were dichotomized (disagree: “does not apply” and “applies somewhat”; agree: “applies mostly” and “totally applies”).

Statement	Agreement, n (%)
I believe that my participation in SaveMySkin will have a positive effect on my well-being.	118 (88.7)
I believe that I will feel supported by SaveMySkin.	119 (89.5)
The effort for the participation in SaveMySkin seems low to me.	86 (64.7)
My motivation to participate in SaveMySkin is high.	121 (91.0)
In general, I have a positive attitude toward communication technologies (eg, computer, mobile phone, internet).	125 (94.0)
Without SaveMySkin, I am sufficiently supplied with health care offers.	32 (24.1)
I want to participate in SaveMySkin because I believe participation would be helpful for me.	130 (97.7)
I believe that I would benefit from participation in SaveMySkin.	131 (98.5)
I would like to participate in SaveMySkin because health care services are insufficient.	83 (62.4)
Other reasons for your participation.	32 (24.1)

**Table 4:** Efficacy (linear mixed-effects models) of the intervention.

Variable			Estimate		95% CI		*t* test (*df*)		*P* value	Cohen *d*
**SPS-R^a^ total score**										
	Time	–1.17		–2.02, –0.33		–2.73 (204.50)		.007		—^b^
	Group	0.77		–0.58, 2.13		1.13 (161.78)		.26		—
	Time × group	–1.59		–2.83, –0.34		–2.50 (203.88)		.01		0.67
**SPS-R Symptom Severity**										
	Time	–0.73		–1.17, –0.29		–3.24 (198.83)		.001		—
	Group	0.60		–0.09, 1.30		1.71 (155.82)		.09		—
	Time × group	–1.01		–1.67, –0.36		–3.06 (197.60)		.003		0.79
**SPS-R Impairment**										
	Time	–0.44		–0.97, 0.09		–1.64 (206.48)		.10		—
	Group	0.19		–0.71, 1.09		0.42 (172.50)		.67		—
	Time × group	–0.57		–1.35, 0.21		–1.44 (206.31)		.15		0.4
**KPD-38^c^ total score**										
	Time	0.02		–0.08, 0.11		0.31 (202.04)		.76		—
	Group	0.02		–0.16, 0.20		0.22 (166.86)		.83		—
	Time × group	–0.05		–0.20, 0.09		–0.72 (200.85)		.47		0.2
**Skin Picking Impact Scale**										
	Time	–0.16		–1.99, 1.68		–0.17 (204.78)		.87		—
	Group	–0.87		–4.14, 2.40		–0.52 (171.56)		.60		—
	Time × group	–1.82		–4.52, 0.87		–1.34 (203.91)		.18		0.37
**MIDAS** ^d^ **focused skin picking**										
	Time	0.20		–0.68, 1.07		0.44 (205.10)		.66		—
	Group	0.15		–1.38, 1.69		0.20 (174.80)		.84		—
	Time × group	–0.60		–1.89, 0.68		–0.93 (204.56)		.35		0.26
**MIDAS automatic skin picking**										
	Time	0.06		–0.71, 0.84		0.17 (203.64)		.87		—
	Group	–0.32		–1.75, 1.10		–0.45 (164.29)		.65		—
	Time × group	–0.41		–1.55, 0.73		–0.71 (202.19)		.48		0.19

^a^SPS-R: Skin Picking Scale–Revised.

^b^Not applicable.

^c^KPD-38: Clinical Psychological Diagnosis System–38.

^d^MIDAS: Milwaukee Inventory for the Dimensions of Adult Skin Picking.

### User Satisfaction

All results regarding user satisfaction refer to the subsample of the intervention group that completed the entire post-assessment questionnaire (38/64).

#### Overall Satisfaction

**Figure 2:**
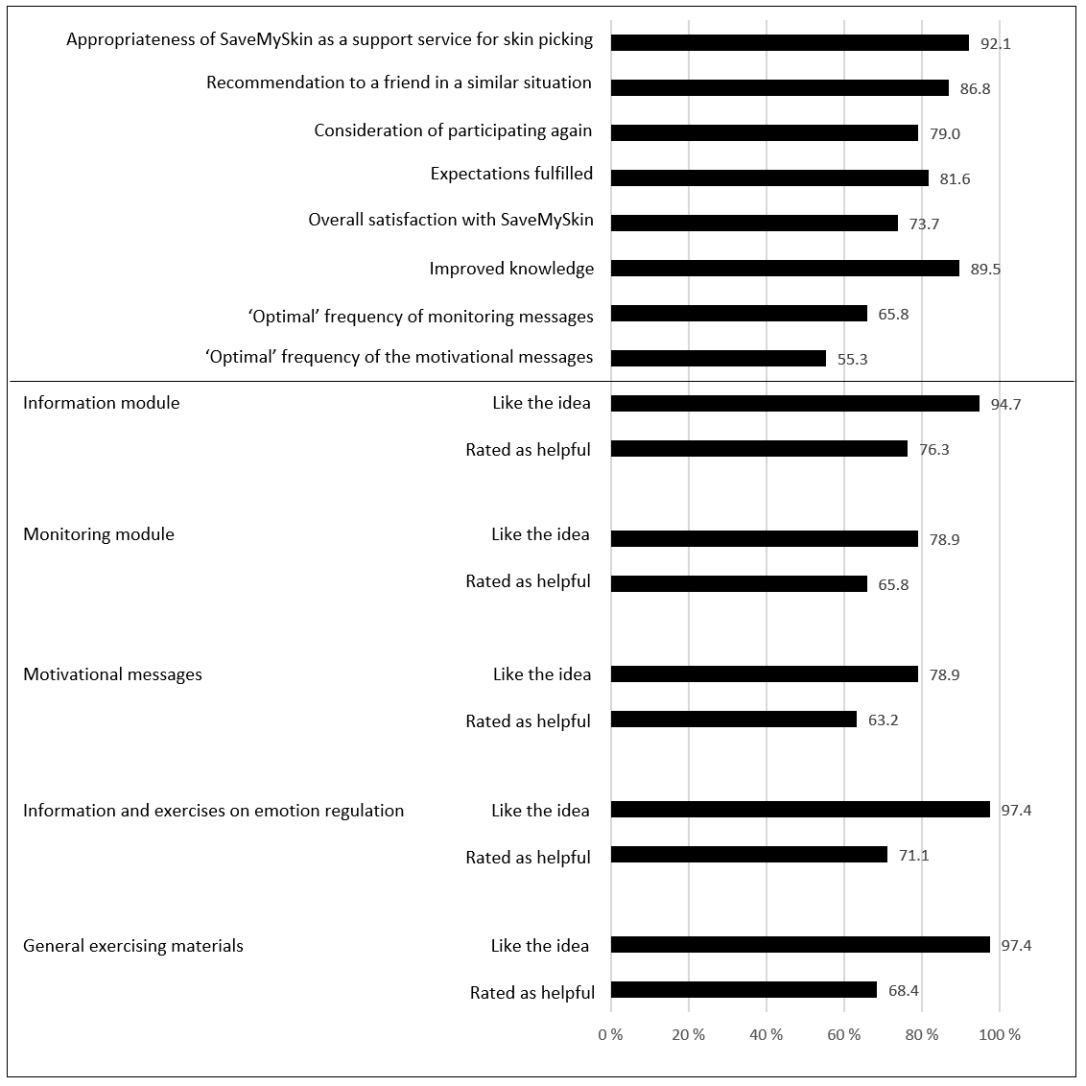
User satisfaction and evaluation of modules.

Almost all participants agreed that the program is an appropriate and supportive offering for individuals with skin picking and that they would recommend it to a friend in a similar situation. Most participants also agreed that the program met their expectations and improved their knowledge on skin picking (Figure 2). The length of the intervention was rated as optimal by 39% (15/38) and as too short by 42% (16/38). Only 18% (7/38) thought it was too long.

#### Evaluation of Modules

More than 94% of the t3 completers in the intervention group (36/38, 95%) liked the idea that information materials, information and exercises about emotion regulation, and additional exercises on several topics were part of the program. The idea of a daily motivational message and monitoring, and inclusion of all three chat types (psychological individual chat, psychological group chat, dermatological group chat) were also positively evaluated by more than three-quarters of the sample (29-34/38, 76% to 89%). Most participants evaluated the different SaveMySkin modules as helpful. The helpfulness of the chat module was not evaluated by most of the participants (82% to 87% for the different chats), since only a very small percentage used the chats. It should be noted that evaluation of the single modules contains up to n=4 values in the category “not able to evaluate” (4/38, 11%). Details on the evaluation of program modules are presented in Figure 2.

### Adherence and Program Use

#### Monitoring

On average, participants completed 25.81 (SD 26.96; range 0-81) out of 84 daily monitoring assessments. Three-quarters of the sample (48/64, 75%) completed more than 3 monitorings, 50% (32/64) answered more than 9, and 25% (16/64) completed more than 45 monitoring questionnaires. In sum, 47% (30/64) participated in the monitoring at least once a week and 42% (27/64) participated at least twice a week.

#### Chat

Five individual chat sessions were booked and carried out (4/64 participants, 6%). The psychological group chat was used by 3 participants (5%) and the dermatological group chat by 6 individuals (9%), who participated up to 3 times in one of the chat types. Participants were asked why they had not used the chat (33/64). The most common answers were “I don’t really know why I didn’t use the chat” (20/33, 61%), “I had no need because I could seek advice somewhere else (eg, psychotherapy)” (13/33, 39%), and “I had no need because I felt good” (12/33, 36%).

Other response options were “I didn’t really know what to expect” (10/33, 30%), “I couldn’t imagine that an internet chat would be helpful in this topic” (8/33, 24%), “It was too much effort for me to book an appointment” (7/33, 21%), “I didn’t know about this option” (5/33, 15%), “I was scared that I am technically not fit enough (eg, cannot type fast enough)” (3/33, 9%), and “Other reasons” (11/33, 33%; most often time conflicts or no time [6/11]).

#### Log-Ins and Views

On average, participants logged in 6.42 (SD 10.15) times (median 3; range 1-67). The average number of page views per user across the following 4 basic modules was 29.31 (SD 42.04; Information: mean 8.17 [SD 11.92]; Skills: mean 11.52 [SD 20.84]; Tools: mean 5.59 [SD 8.09], Emotions: mean 4.03 [SD 6.16]). These modules contain 35 pages in total. Answering the daily monitoring questionnaire was not counted as a log-in.

### Effects on Secondary Outcomes

No statistically significant time × group interactions occurred for secondary outcome variables (Table 4).

### Help-Seeking Behavior During Participation

At the time of post-assessment, 17% (8/48) of the control group and 18% (7/38) of the intervention group who completed the t3 questionnaire indicated that they had used professional help due to skin picking in the last 12 weeks. In the intervention group, 24% (9/38) indicated that they were planning to use professional help; in the control group, 15% (7/48) were planning to seek professional help.

## Discussion

### Principal Findings

Skin picking disorder is associated with psychological distress, impairment in social life, and medical risks, but currently individuals with skin picking rarely receive the required professional support due to an insufficient health care supply. Our study investigated attitudes and expectations toward an internet-based self-help program for skin picking as well as user satisfaction and effects on skin picking severity and impairment.

Full recruitment of the initially targeted sample size for this pilot trial was rapidly achieved, indicating a high willingness to participate in an internet-based intervention for skin picking. Randomization resulted in two comparable study groups that only differed marginally in the SPS-R subscale Symptom Severity.

Participants were highly motivated and expected increased well-being and a feeling of support provided by the program. Such positive expectations are known to be an important factor contributing to intervention effects [40]. Flexibility in terms of time and location, expertise related to skin picking, and a lack of other health care options were further reasons for participating. Almost all t3 completers in the control group requested access to the intervention (96%) after the waiting period of 12 weeks, indicating a persistent motivation to use the intervention.

The majority of those in the intervention group who completed the post-assessment reported a high satisfaction with the modules included in SaveMySkin and the program in general (eg, appropriateness, length, recommendation to others). On average, participants completed more than 2 monitorings per week, suggesting that daily monitorings might be too frequent.

The intervention group yielded substantial reductions in skin picking severity (SPS-R total score) and specifically in the subscale Symptom Severity compared with the control group. The size of these effects (*d*=0.67 and *d*=0.79) is comparable to the overall effect of behavioral treatments for skin picking disorder reported in a meta-analysis (standardized mean difference 0.68) [20]. The analyses did not confirm meaningful differences between groups regarding improvements in skin picking–related impairment measured via the SPS-R and SPIS. Given the rather short time period covered in the trial, this result is not surprising since skin picking–related impairment (eg, impaired self-esteem, avoidance, skin damage) may only improve slowly, even if skin picking frequency and intensity are improved. Furthermore, some medical consequences, especially scars, often need to be considered as permanent. The short study period may also be responsible for the lack of effects on general psychological impairment and different dimensions of skin picking (focused vs automatic) in our study. Dimensions of skin picking were assessed with regard to habitual but not necessarily current patterns (eg, “I am usually not aware of picking my skin during the picking episode”), so potential changes might not be reflected properly. Also, sensitivity to change has yet to be explored for this assessment instrument (MIDAS). Apart from the MIDAS, the applied instruments proved appropriate for interventional studies. As the primary outcome measure, the SPS-R proved to be sensitive to change. This is of special importance for subsequent studies, given the lack of interventional studies on skin picking disorder and the associated uncertainty about the adequate measurement of intervention effects.

Concerning use of the self-help program, it turned out that the chat module was used only rarely, even though most participants (more than 76%) liked the idea that different chat modules were included in the program. Given that chatting is not an obligatory key element of the program but an optional offer for those who feel the need for personal counseling, the low chat use is not concerning. Rather, it is in accordance with previous research suggesting that a considerable number of users in online communities do not actively produce content (eg, posting in a forum) but rather read and browse through the platform [41]. More than half of the participants who did not use the chat indicated that they were not sure why. Potential underlying reasons could, for example, be insecurity and shyness when talking with others about this very personal topic, even if it is online, or a reluctance to commit to chat participation at a certain date and time. Even though various reasons led to a rather infrequent use of the module, it seems to be important to keep the chat as part of the program because most participants liked the idea that the different chats were included. The module may not be used by many participants, but it could nevertheless be very important for some individuals, especially for severely impaired individuals without access to other support.

### Limitations and Implications for Future Research

This study included several limitations, which should be considered in the interpretation of results. First, all assessments were conducted online, and inclusion relied exclusively on self-reported data, so internal validity and generalizability to a larger clinical population might be compromised. Another shortcoming results from the rather low response rate at post-assessment. The lack of data from approximately one-third of the sample limits the validity of our findings, since it remains unclear how satisfied the nonresponders were and how they changed in symptomatology. Additionally, our study did not investigate the stability of effects, as it focused on the classic aims of a pilot study and therefore did not include extended follow-up assessments. However, the results of this study clearly demonstrated the feasibility of an internet-based intervention in the target group. Furthermore, the study provided preliminary evidence for the efficacy of the intervention. Subsequent research should therefore investigate the efficacy and cost effectiveness of SaveMySkin in a fully powered RCT. This trial should apply a sequential enrollment procedure including a clinician-rated assessment of psychiatric conditions and a dermatological assessment and documentation of the skin status. Moreover, the study period should be expanded to assess the stability of intervention effects and explore potential long-term effects on impairment.

The program use in our study and user feedback suggest minor adaptions of the program, which will be implemented to ensure that users’ needs are met even better by the provided support. The dermatological group chat will be replaced by a forum where participants can ask dermatologists for advice. This asynchronous form of communications seems to fit user behavior better, since the forum can be accessed any time and does not require meeting a certain appointment. Additionally, the self-management module will be reorganized into topical sessions that will guide participants through the process in a more structured manner. This adjustment was recommended by several users. It may also improve program use, since users receive more guidance and make visible progress as they move from one session to the next. Moreover, visibility of progress will be improved by including a new module providing visual feedback on daily monitoring data (eg, graph displaying changes in symptom severity) and completed exercises within sessions. Additionally, following user recommendations, the daily monitoring messages will no longer be delivered via email but via mobile messaging technology (eg, short message service) in order to facilitate more immediate participation. These adjustments are expected to improve user satisfaction and the extent of program use and may ultimately further enhance the efficacy of the intervention. In a subsequent efficacy study, the impact of certain program modules should be analyzed in more detail by exploring associations between program use (especially within the monitoring module) and changes in skin picking symptomatology.

The pilot study derived important knowledge on the feasibility and adequacy of an internet-based intervention for skin picking disorder and therefore provides essential information for subsequent research. We are aware that in general the intervention effects observed in a pilot trial have only limited value for guiding the preparation of a subsequent RCT (eg, in terms of power calculations) [42]. Therefore, the pilot results should be considered as highly promising but preliminary evidence that should be interpreted with adequate caution.

### Conclusions

To our knowledge, this is the first study to comprehensively investigate an internet-based self-help intervention for skin picking disorder in an RCT. This pilot study showed that SaveMySkin seems to be a welcomed and suitable program for individuals with skin picking that can induce substantial improvements in symptomatology. The results of the pilot trial will be used to design a subsequent study on the efficacy and cost effectiveness of SaveMySkin, which may be a beneficial complement to conventional health care for skin picking disorder.

## Appendix

Multimedia Appendix 1 CONSORT‐EHEALTH checklist (V 1.6.1).

## Abbreviations

CBT: cognitive-behavioral therapy
DSM-5: Diagnostic and Statistical Manual of Mental Disorders, 5th Edition
KPD-38: Clinical Psychological Diagnosis System–38
MIDAS: Milwaukee Inventory for the Dimensions of Adult Skin Picking
RCT: randomized controlled trial
SPIS: Skin Picking Impact Scale
SPS-R: Skin Picking Scale–Revised
